# Bayesian bias adjustments of the lung cancer SMR in a cohort of German carbon black production workers

**DOI:** 10.1186/1745-6673-5-23

**Published:** 2010-08-11

**Authors:** Peter Morfeld, Robert J McCunney

**Affiliations:** 1 Institute for Occupational Medicine of Cologne University/Germany; 2 Institute for Occupational Epidemiology and Risk Assessment of Evonik Industries, Essen/Germany; 3 Department of Biological Engeneering, Massachusetts Institute of Technology, Boston/USA

## Abstract

**Background:**

A German cohort study on 1,528 carbon black production workers estimated an elevated lung cancer SMR ranging from 1.8-2.2 depending on the reference population. No positive trends with carbon black exposures were noted in the analyses. A nested case control study, however, identified smoking and previous exposures to known carcinogens, such as crystalline silica, received prior to work in the carbon black industry as important risk factors.

We used a Bayesian procedure to adjust the SMR, based on a prior of seven independent parameter distributions describing smoking behaviour and crystalline silica dust exposure (as indicator of a group of correlated carcinogen exposures received previously) in the cohort and population as well as the strength of the relationship of these factors with lung cancer mortality. We implemented the approach by Markov Chain Monte Carlo Methods (MCMC) programmed in R, a statistical computing system freely available on the internet, and we provide the program code.

**Results:**

When putting a flat prior to the SMR a Markov chain of length 1,000,000 returned a median posterior SMR estimate (that is, the adjusted SMR) in the range between 1.32 (95% posterior interval: 0.7, 2.1) and 1.00 (0.2, 3.3) depending on the method of assessing previous exposures.

**Conclusions:**

Bayesian bias adjustment is an excellent tool to effectively combine data about confounders from different sources. The usually calculated lung cancer SMR statistic in a cohort of carbon black workers overestimated effect and precision when compared with the Bayesian results. Quantitative bias adjustment should become a regular tool in occupational epidemiology to address narrative discussions of potential distortions.

## Background

Carbon black is a powdered form of elemental carbon that is manufactured by the controlled vapor-phase pyrolysis of hydrocarbons. Preferential raw materials for most carbon black production processes are feedstock oils that contain a high content of aromatic hydrocarbons. Over 90% of the world's carbon black production is used for the reinforcement of rubber; about two thirds are used for tires and one third for the production of technical rubber articles.

Car tires contain approximately 30% to 35% of carbon blacks of different types. The remaining world production of carbon black is used for printing inks, colours and lacquers, stabilizers for synthetics, and in the electrical industry [1]. Currently, greater than 95% of worldwide carbon black production is *via *the oil furnace black process [2]. Different grades of carbon black are typically produced by using different reactor designs and by varying the reactor temperatures and/or residence times [3].

The most recent evaluation of possible human cancer risks due to carbon black exposure was performed by an IARC (International Agency for Research on Cancer) Working Group in February 2006 [4]. The Working Group identified lung cancer as the most important endpoint to consider and exposures to workers at carbon black production sites as the most relevant for an evaluation of risk. The group concluded that the human evidence for carcinogenicity was *inadequate*. (IARC, overall Group 2B)

Among the key studies evaluated by IARC [4] was a German investigation of 1,528 carbon black production workers[5-7]. Based on 50 observed cases a lung cancer SMR (standardized mortality ratio) of 2.18 (0.95-CI: 1.61, 2.87; national reference rates from West Germany; CI = confidence interval) or 1.83 (0.95-CI: 1.34, 2.39; state reference rates from North-Rhine Westphalia) was estimated. Positive trends with carbon black exposures were not observed in internal dose-response analyses [6,7]. However, a nested case-control study [8] identified smoking and previous exposures to known carcinogens prior to work at the carbon black plant as important risk factors. Due to correlations between previous exposures to carcinogens, crystalline silica exposure was used as a surrogate for the group of occupational confounders experienced prior to work at the carbon black plant (see Büchte and co-workers [8] for details). A simple sensitivity analysis concluded that these two factors (smoking and previous exposures) may explain the major part of the excess risk in lung cancer reported in the original cohort analysis [5]. The IARC working group raised concerns as to whether the simple sensitivity analysis was appropriate for adjustment since the findings were difficult to interpret. We thus now present results from a Bayesian bias adjustment that addresses deficiencies of the simple sensitivity analysis.

Customarily, confidence intervals estimate random error, not other sources of uncertainty, such as confounding, selection bias and measurement error. To address this additional uncertainty of an effect measure simple sensitivity analyses, Monte Carlo sensitivity analyses (Probability Sensitivity Analyses) or Bayesian analyses can be used - but Bayesian analyses appear to come with the stronger rationale because the only formal statistical interpretation available for Monte Carlo simulation approaches is Bayesian [9,10]. In addition, practical advantages exist when the analyst follows the Bayesian approach [11]. In retrospective mortality studies, such as the German carbon black cohort described above, information on smoking and previous exposures is either lacking or incomplete. By including the limited information available on smoking and previous exposures from a case-control study [8] in a Bayesian framework quantitative estimates of the uncertainty of the SMR as a result of confounding can be determined. We use the carbon black example to apply and illustrate this method. Details of the procedure and explanations of the Bayesian approach are given in the Methods section. We implemented the approach by Markov Chain Monte Carlo Methods (MCMC) programmed in R, a statistical computing system freely available on the internet. We provide the program code in an Additional File. This may help a reader to understand the procedure in detail.

## Methods

The cohort consisted of all male German blue-collar workers who were continuously employed at the carbon black production plant for at least one year between Jan 1^st ^1960 and Dec 31^st ^1998 and (1) whose mortality could be followed beyond 1975; and (2) if deceased, died from a known cause of death [6]. The cohort consisted of 1528 carbon black workers and 25,681 person-years; 7 subjects with unknown cause of death were excluded. In this cohort, 50 subjects died of lung cancer. This Bayesian analysis focused on the SMR findings of the national reference rates to avoid over-adjustment due to differences in smoking behaviour between West-Germany and the state North-Rhine Westphalia. We therefore based all adjustment procedures on the higher lung cancer SMR estimate of 2.18 (0.95-CI: 1.61, 2.87) reported in the first cohort analysis [6].

The Bayesian adjustment procedure followed an outline proposed by Steenland and Greenland [12], including how to structure a Bayesian model of unmeasured or only partly measured confounders, and how to derive an adjusted posterior SMR after applying all available background information. A posterior SMR is a term used in Bayesian analysis that includes both, a priori knowledge about the parameter that models the unmeasured or partly measured confounding and the standard frequentist statistical assessment.

Frequentist methodology assumes that parameters are fixed and that the observed data were realized from a probability distribution given the parameters. This distribution is described by the likelihood function, P(data | parameters), i.e., the probability of the data given the parameters. Frequentists usually base their conclusions only on this function and the observed data. In contrast, a central idea of Bayesian thinking is that parameters are uncertain. First, this uncertainty obviously exists at the beginning of all discussions and research. Second, this uncertainty about parameters cannot be removed by new data totally - but the degree of uncertainty can be modified in the light of new data. Bayesian theory quantifies the knowledge and uncertainty we begin with in terms of a prior distribution of the parameters, P (parameters). In subjective Bayesian theory this first input to the analysis describes how the analyst would bet about the parameters if the data under analysis were ignored. The likelihood function - as used by the frequentists - is the second input to the Bayesian analysis. It describes the probability the analyst would assign to the observed data given the parameters. How to move forward from here? Basic rules of probability theory imply the Bayesian theorem. This theorem says

P(parameters|data)=P(data|parameters)​​ P(parameters)/P(data).

The Bayesian theorem states how we should modify our knowledge and degree of uncertainty about the parameters after we have analyzed the observed data. The goal of the analysis is to calculate how we should bet about the parameters after the data was observed and analyzed. Therefore, we are interested in P(parameters | data), that is the posterior distribution of the parameters. The factor 1/P(data) is often called the proportionality factor and this factor links the posterior with the product of likelihood and prior. The parameters that occur in the problem may be split into target parameters and bias parameters. What we are really interested in are the target parameters, like the SMR. But bias parameters may have distorted the data we observed to learn about the target. The distribution of both kinds of parameters can be updated with the help of the Bayesian theorem. The posterior target parameters, we are mainly interested in, are the adjusted target parameters taking the distribution of bias parameters, our prior knowledge about the target parameters and the observed data into account. In summary, Bayesian bias analysis offers an analysis that adjusts the SMR (= target parameter) and estimates the uncertainty of the SMR by including a quantitative assessment of the effect of bias, and in particular, confounding, on the results. We provide a glossary of key terms used in this article in Additional File 1.

How are results reported? The central tendency ("point estimate") is often described by the median of the posterior distribution (e.g., [12]) because the median is not as vulnerable to skewness and extreme values in the empirical posterior distribution as the mean [13]. The degree of uncertainty ("interval estimate") is often reported as the central 95% region of the posterior distribution and is called 95% posterior interval or 95% Bayesian interval ([9], p. 332, 379) or 95% highest density region or 95% credible interval ([14], p.49). The latter name points to an important distinction: whereas the 95% posterior interval can be validly interpreted like "Given these prior, likelihood, and data we would be 95% certain that the parameter is in this interval." The conventional 95% confidence interval has no such appealing interpretation. The following difficult statement is logically justified as an interpretation of conventional 95% confidence intervals given a probability of 5% is accepted as an indicator of "improbable": "If these data had been generated from a randomized trial with no drop-out or measurement error, these results would be improbable were the null true." ([9], p. 333). Note that Rothman and colleagues added "but because they were not so generated we can say little of their actual significance". Indeed, in observational epidemiology there is no such data generating mechanism at work. Thus, the Bayesian approach offers an advantage because interval estimates can be interpreted in a "natural" way.

As an introduction into Bayesian perspectives and procedures, we refer to papers by Greenland [15,16] and also suggest reading more detailed overviews of Bayesian applications and philosophy [9,14,17,18]. An easy to read but profound introduction into Bayesian statistics was given by Greenland in chapter 18 of [9]. A good overview of bias analysis in epidemiology was written by Greenland and Lash (chapter 19 of [9]). An application of Bayesian techniques in bias adjustment via data augmentation and missing data methods was explained and exercised in Greenland 2009 [11].

Although we followed the outline proposed by Steenland and Greenland 2004 [12] some notable differences exist. An important extension in this analysis is that it shows how to deal with more than just one uncontrolled cause of bias. Steenland and Greenland 2004 [12] adjusted for uncontrolled smoking with the help of Bayesian methods. Here we adjusted for two bias factors, smoking and prior exposures experienced before being hired at the carbon black plant. However, Steenland and Greenland 2004 [12] were able to use a three-level smoking variable whereas we could only rely on binary coded smoking data. More importantly, we examined the impact of different prior explications, in particular non-flat priors and of correlations between prior parameters, which are topics not covered by Steenland and Greenland 2004 [12]. For more details see the discussion section of this report.

The SMR as obtained in mortality studies is customarily adjusted only for age, gender and calendar time. Confounding, such as cigarette smoking is not addressed. Thus, the SMR is potentially biased. To adjust the SMR for partly measured potential confounders like smoking, we developed a likelihood of the outcome data. In this study, the outcome data were simply the number of observed cases (observed = 50 lung cancer deaths). This number of observed cases depends on three values: a) the number of expected cases, calculated with the help of reference rates (expected = 22.9 lung cancer deaths), b) the unbiased SMR_true _and c) the degree of bias.

Under usual assumptions [19] (customary frequentist statistic) we can write

$$\text{observed}\sim \text{Poi}(\text{expected}*{\text{SMR}}_{\text{true}}*\text{bias}),$$

Where Poi(λ) denotes the Poisson distribution with parameter λ and * denotes multiplication.

This specifies the likelihood P(observed | expected, SMR, bias). [Here and in the following we drop the index "true" for the sake of simplicity.]

In our case we assumed that the bias stems from two sources (smoking and previous exposures, see Background section) and can be written [20]

$$\text{bias}={\text{bias}}_{\text{smoke}}*{\text{bias}}_{\text{prev}}.$$

To explicate the likelihood we had to quantify the bias components bias_smoke _and bias_prev_. We supposed that bias_smoke _depends on three prior parameters

▪ prop_smoke, pop _: proportion of smokers/ex-smokers in the general population

▪ prop_smoke, coh _: proportion of smokers/ex-smokers in the carbon black cohort

▪ OR_smoke _: odds ratio of lung cancer mortality for smokers/ex-smokers vs. never smokers

and that the degree of bias could therefore be estimated as

$${\text{bias}}_{\text{smoke}}=\frac{{\text{prop}}_{\text{smoke,coh}}{\text{*OR}}_{\text{smoke}}+{\text{1-prop}}_{\text{smoke,coh}}}{{\text{prop}}_{\text{smoke,pop}}{\text{*OR}}_{\text{smoke}}+{\text{1-prop}}_{\text{smoke,pop}}}.$$

The derivation of this formula is given in Additional File 2. It is based on concepts developed and applied by Cornfield et al. 1959 [21] (reprinted as Cornfield et al. 2009 [22]), Bross 1966 [23], Yanagawa 1984 [24] or Axelson and Steenland 1988 [25].

A similar argument can be applied to estimate the bias due to previous exposures (bias_prev_.) It depends on the three prior parameters

▪ prop_prev, pop _: proportion of subjects occupationally exposed to crystalline silica in the general population

▪ prop_prev, coh _: proportion of subjects previously exposed to crystalline silica in the carbon black cohort

▪ OR_prev _: odds ratio of lung cancer mortality for previous exposure to crystalline silica

and can be calculated as

$${\text{bias}}_{\text{prev}}=\frac{{\text{prop}}_{\text{prev,coh}}{\text{*OR}}_{\text{prev}}+{\text{1-prop}}_{\text{prev,coh}}}{{\text{prop}}_{\text{prev,pop}}{\text{*OR}}_{\text{prev}}+{\text{1-prop}}_{\text{prev,pop}}}.$$

We derived a prior distribution for the three parameters defining the bias due to differences in the smoking behaviour between cohort and population and we derived a prior distribution for the three parameters defining the bias due to differences in the exposure to crystalline silica dust exposure between cohort and population. This information was incorporated into the likelihood so that the usual frequentist approach was extended by the prior data. Defining and applying a full distribution and not only a point estimate for, say, prop_smoke, coh _has the advantage of taking the uncertainty of this parameter estimate into account whereas this uncertainty, although existing without doubt, is usually ignored in a simple sensitivity analysis [5,26].

Firstly, we derived distributions for the proportion of smokers in the cohort and in the population. We made extensive use of the logit-function because it can be readily applied to approximate distributions of proportions by the Gaussian distribution [12]. The logit-transformation is defined as logit x = log (x/(1-x)) with log denoting the natural logarithm. We use N(μ,σ^2^) to denote the Gaussian distribution with mean μ and variance σ^2^. An approximate distribution of a proportion p can be described as follows [12]: If p_obs _denotes the observed proportion among n subjects and p the random variable realised as p_obs _we use logit (p) ~ N(μ,σ^2^) as an excellent approximation with μ estimated by logit p_obs _and σ estimated by s = (p_obs _(1- p_obs_)n)^(-1/2)^. We applied this formula to data about the smoking prevalence in the cohort. We derived and used two candidates for the distribution of p in the cohort, one based on case-control information [8] about smoking and one based on cohort information [5]. The proportion of subjects acting as controls and classified as smokers or ex-smokers in the nested case-control study group was 84% [8] and the proportion of subjects in the cohort who were classified accordingly was 83.95% [5]. Using these percentages based on 48 control subjects in the case-control study [8] and based on 1180 workers with smoking information in the cohort study [5] we derived the following two alternative priors, both estimating the proportion of smokers in the cohort: (a) logit(0.84) = 1.66, s = (48*0.84*0.16)^(-1/2) ^= 0.394, i.e., logit prop_smoke, ncc _~ N(1.66, 0.394^2^) using nested case-control information, and (b) logit(0.84) = 1.66, s = (1180*0.84*0.16) ^(-1/2) ^= 0.0794, i.e., logit prop_smoke, coh _~ N(1.66, 0.0794^2^) when applying cohort data. Next, we derived an approximate distribution for the proportion of smokers in the population. Given a proportion of 65% smokers among males in West-Germany based on a representative sample of 3450 men [27,28] we calculated for the population logit(0.65) = 0.619, s = (3450*0.65*0.35)^(-1/2) ^= 0.0357 and, therefore, set logit prop_smoke, pop _~ N (0.62, 0.0357^2^) accordingly.

Secondly, we derived a distribution of the effect of smoking on lung cancer mortality. The conditional logistic regression for lung cancer mortality depending on a smoking indicator (active smokers/ex-smokers vs. never smokers) yielded an odds ratio of OR_smoke _= 9.27 (0.95-CI: 1.16, 74.4) when analyzing the nested case-control study [8]. Based on this information we estimated log OR_smoke _= 2.227 with a standard deviation of s_smoke _= log(74.4/1.16)/3.92 = 1.061, the latter calculated from the 95%-confidence interval for OR_smoke _applying a Gaussian approximation to log OR_smoke. _Therefore, we set log OR_smoke _~ N(2.23, 1.06^2^) as the informative prior about the effect of smoking in our cohort. This Gaussian approximation holds because the log OR is identical to the coefficient in the logistic regression model and the coefficient is normally distributed according to maximum likelihood theory [19].

Next, we had to construct a prior distribution for the three parameters defining bias_prev_. Again we made use of the logit-approximation to derive a prior for the proportions of subjects being exposed to silica. And again, as with smoking, we derived two candidates for the distribution of the proportion in the cohort, one based on an application of CAREX [29,30] which is a computer assisted information system for the estimation of the numbers of workers exposed to established and suspected carcinogens and one based on an expert assessment. Büchte and co-workers [8] applied the data of the CAREX system [29,30] to derive automatic estimates of previous exposures within the nested case-control: since 74% of the 88 workers (controls) were identified as previously exposed we got logit (7%) = 1.05 and s = (88*0.74*0.26)^(-1/2) ^= 0.2432. This lead to a prior of logit prop_prev, coh _~ N(1.05, 0.243). This is the "CAREX cohort prior".

A brief description of the CAREX system [29,30] is warranted. CAREX is a computer assisted information system for the estimation of the numbers of workers exposed to established and suspected human carcinogens in the member states of the European Union. This system can be automatically applied to estimate the probability of being exposed to a specific carcinogen. Details of how it was used in this study are given elsewhere [8]. CAREX is based on information about occupational exposure in 1990 to 1993 estimated in two phases. Firstly, estimates were generated on the basis of Finnish labour force data and exposure prevalence estimates from two reference countries (Finland and the United States) which had the most comprehensive data available on exposures to these agents. For selected countries, these estimates were then refined by national experts in view of the perceived exposure patterns in their own countries compared with those of the reference countries.

Blinded to the CAREX system [29] data and to the case-control status, a German occupational-exposure expert independently assessed whether the study members of the case-control study were exposed to occupational carcinogens before being hired at the carbon black plant [8]: since 16% of the 88 workers (controls) were documented as exposed by this expert, we derived logit (16%) = -1.66, s = (88*0.16*0.84)^(-1/2) ^= 0.2912 and therefore got a second prior suggestion: logit_prev, coh _~ N(-1.16, 0.291). This is the "expert cohort prior".

In the next step, we derived an approximate distribution of the percentage of male workers exposed to crystalline silica in the population. Whereas we defined just one prior for the percentage of smokers in the population the situation is more complicated with silica dust exposure. We derived two main candidates for the prior and two further candidates used in an additional sensitivity analysis. Based again on the CAREX system [29] the percentage of male workers occupationally exposed to crystalline silica in the population was estimated as 2.3%. We set logit (2.3%) = -3.74, 0.95-CI: 2.3%/2, 2.3%*2, i.e., s = 0.3536 and therefore logit_prev, pop _~ N(-3.74, 0.3536). This is the "CAREX population prior". Here we assumed implicitly that the CAREX estimate is unstable by a factor of two. Since the German expert did not assess the degree of crystalline silica exposure of the male population, we proceeded as follows. The expert documented 16% of the controls being exposed but the CAREX system [29] estimated 74%. We used the ratio of these percentages to adjust the CAREX estimate of the population prevalence accordingly: 16/74*2.3% = 0.5%, and we set logit (0.5%) = -5.30, 0.95-CI: 0.5%/2, 0.5%*2, i.e., s = 0.3536 which leads to logit_prev, pop _~ N(-5.30, 0.3536). This is the "expert population prior". This was used as the main population prior in the calculation based on the German expert's data. Because this prior appears to be difficult to justify as a reliable description of the crystalline silica dust exposure distribution in the population (based on the expert's opinion) we repeated the analysis while assuming a prior with a larger spread (corresponding to a factor of 5): logit_prev, pop _~ N(-5.30, 0.8211). Note that log(5)/1.96 = 0.8211. In addition we used a prior with an expectation equal to the "CAREX population prior" but accompanied with a larger spread (again corresponding to a factor of 5): logit_prev, pop _~ N(-3.74, 0.8211). These different priors (one main and two further candidate "expert population priors") were used to study the sensitivity of the results due to our missing knowledge about the prevalence of crystalline silica dust exposure in the population if the expert had estimated it.

Finally, we needed an estimate of the effect of previous silica dust exposure on lung cancer risk. Again we derived two explications, one based on the CAREX [29,30] data and the other based on the expert's assessment. Analyzing the nested case-control study by conditional logistic regression yielded a smoking adjusted OR = 2.1 (0.95-CI = 0.39, 11.2) for the CAREX based indicator of being previously exposed to crystalline silica [8]. This lead to log OR = 0.74, s = log(11.2/0.39)/3.92 = 0.8565 and, thus, we derived as the prior log OR_prev, coh _~ N(0.74, 0.857). This is the "CAREX effect prior". Based on the German expert's data, the OR for previous exposures was estimated as 5.06 (0.95-CI= 1.68, 15.27). Applying a conservative correction for smoking [6,8] we got OR = 5.06*2.04/3.28 = 3.14, i..e., log OR = log(5.06*2.04/3.28) = 1.146, s = log(15.27/1.68)/3.92 = 0.5632 and set log OR_prev, pop _~ N(1.15, 0.563) as the prior. This is the "expert effect prior".

Because we did not think it appropriate to rely on a single overall prior that may not be able to represent all available prior knowledge, we derived instead different explications of bias_smoke _and bias_prev _as outlined above and used these explications in sensible combinations to derive four main Bayesian analyses. The structure of this approach is summarized in Table 1.

**Table 1:** Gaussian prior distributions (mean μ and standard deviation σ) applied in the four analyses.

	Analysis							
	
	CAREX				Expert			
	
	smoking cohort		smoking case-control		smoking cohort		smoking case-control	
	
	1		2		3		4	
	μ	σ	μ	σ	μ	σ	μ	σ
Effect								
log OR _smoke_	2.23	1.06	2.23	1.06	2.23	1.06	2.23	1.06
log OR _prev_	0.74	0.857	0.74	0.857	1.15	0.563	1.15	0.563
								
Proportions								
logit prop _smoke, pop_	0.62	0.0357	0.62	0.0357	0.62	0.0357	0.62	0.0357
logit prop _smoke, coh_	1.66	0.0794	1.66	0.394	1.66	0.0794	1.66	0.394
								
logit prop _prev, pop_	-3.74	0.366	-3.74	0.366	-5.30	0.356	-5.30	0.356
logit prop _prev, coh_	1.05	0.243	1.05	0.243	-1.16	0.291	-1.16	0.291

One effect specification was used throughout to describe the prior for smoking (log OR_smoke_). Two effect specifications were applied to estimate the effect of previous exposures (log OR_prev_): one was based on CAREX data (Analyses 1 and 2) and a second based on data assessed by a German expert (Analyses 3 and 4). The proportion of male smokers in the population was estimated in all analyses by a representative sample from the male population (logit prop_smoke, pop_). Two estimates were derived for the cohort percentage (logit prop_smoke, coh_): one based on cohort data (Analyses 1 and 3) and a second based on case-control information (Analyses 2 and 4). The prevalence of previous occupational exposure to crystalline silica (logit prop_prev, pop_) was estimated by the CAREX system (Analyses 1 and 2) or adapted to fit to the German's expert data (Analyses 3 and 4). The proportion of silica exposed males in the cohort (logit prop_prev, coh_) was derived from CAREX data (Analyses 1 and 2) or from assessments of the German expert (Analyses 3 and 4). For the SMR we always used a flat prior: log SMR ~ N(0,10^8^).

Given the likelihood of the data P (observed | expected, SMR, bias) as explicated we calculated an adjusted (posterior) SMR by Bayes' theorem after inserting the bias priors derived above. However, to apply the theorem, it was also necessary to insert an appropriate prior distribution for the true SMR.

We followed Steenland and Greenland [12] and used an uninformative, flat prior P (SMR) specified by

$$\log\text{SMR}\sim \text{N}{\left(0,10\right)}^{8}.$$

Here log denotes again the natural logarithm and N(μ,σ^2^) the Gaussian distribution with mean μ and variance σ^2^.

The adjusted SMR is given by the posterior distribution P (SMR|observed) that now can be derived with the help of Bayes' theorem as

P(SMR,bias|observed)=factor*P(observed|expected,SMR,bias)*P(SMR,bias).

Integrating over the bias in P (SMR, bias | observed) gives the marginal distribution of the posterior SMR we were interested in mainly. Unfortunately, the calculation is often difficult and usually no closed analytical solution in elementary functions exists. In particular, the proportionality factor is difficult to determine. However, a numerical solution is possible using a Markov Chain Monte Carlo (MCMC) simulation approach [31]. In particular, the posterior can be estimated by MCMC without knowing or calculating the standardizing factor. Concept and proof of this approach were developed and given by Metropolis and co-workers [32] and Hastings [33]. Here we applied a Metropolis' Gaussian random walk generator following the implementation instructions given by Newman [34]. All prior distributions were assumed to be independent. We chose a burn-in phase of 50,000 cycles and evaluated the Markov chain over a length of 1,000,000. We tuned the random walk parameters (σ's of the Gaussian proposal distribution) in such a way that the acceptance rate was between 20% and 40% for all parameters estimated [31].

We plotted the trace for all parameters as simple diagnostic tools informing about goodness of sampler convergence. An introduction to trace plots is given in the Statistical Analysis System (SAS) documentation [35].

All analyses were done with the R package [36]. The program doing Analysis 1 (see Table 1 for definition) is given in Additional File 3.

## Results

**Figure 1:**
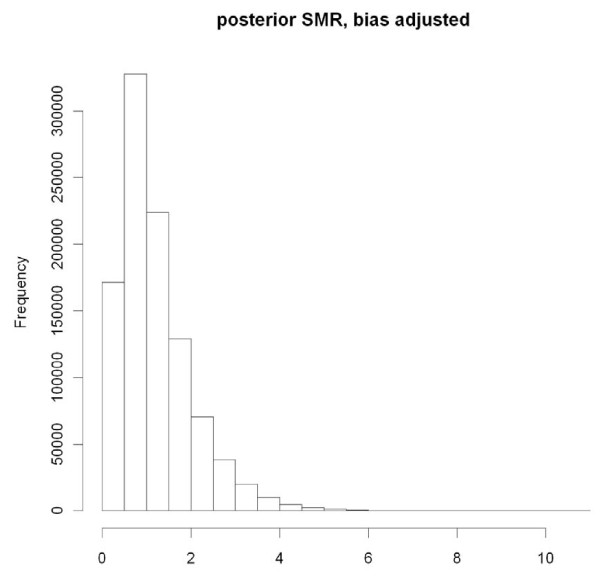
**Distribution of the posterior lung cancer SMR based an Analysis 1 (see Table 1): previous exposures estimated by the CAREX method, smoking estimates based on cohort data**. Results from an MCMC random walk of length 1,000,000 (Metropolis sampler). The x-axis stretches to the maximum of 10.7. Other characteristics of this empirical posterior distribution are given in Table 2.

The distribution of the adjusted lung cancer SMR produced by Analysis 1 (see Table 1 for definition) is shown in Figure 1. The MCMC random walk generated a wide spread of posterior SMR (adjusted SMR) values with half of the estimates below the reference point of 1.

An overview of the results from all four analyses is given in Table 2.

**Table 2:** Characteristic statistics of the posterior lung cancer SMR distribution, i.e., the distribution of the bias adjusted SMR.

	Analysis			
	
	CAREX		Expert	
	
	smoking cohort	smoking case-control	smoking cohort	smoking case-control
	
	1	2	3	4
SMR, posterior				
median	1.00	1.01	1.32	1.32
arithmetic mean	1.21	1.22	1.33	1.34
standard deviation	0.82	0.83	0.34	0.35
2.5%-fractile	0.24	0.25	0.70	0.70
97.5%-fractile	3.31	3.37	2.04	2.07

Findings are reported according to the four analyses described in Table 1.

The number of significant digits displayed is for comparison purposes only. The data set is not of sufficient size to support this accuracy.

Results from MCMC random walks (Metropolis sampler) of length 1,000,000.

Analysis 2 resulted in almost exactly the same findings from Analysis 1. Very similar results were produced also by Analyses 3 and 4. Therefore, it made no relevant difference whether the bias adjustment was based on smoking data from the cohort (Analyses 1 and 3) or on the information gained from the nested case-control study (Analyses 2 and 4). [This similarity of findings is somewhat expected because the competing analyses involve inflating the prior variance of the proportion of smokers in the cohort This should not affect results substantially because it is the prior mean of the bias parameters that dictates the magnitude of unmeasured confounding.] Lower posterior SMRs were calculated when using the automatic previous exposure assessment by the CAREX approach (Analysis 1 and 2): median adjusted SMRs were found at 1, arithmetic averages at about 1.2. The posterior lung cancer SMR estimates showed a median and mean of about 1.3 when using expert data. The analysis based on the CAREX data produced a wider range of bias adjusted estimates (95% posterior interval: 0.2, 3.4) than the findings from the Bayesian analyses when applying the expert's assessment (95% posterior interval: 0.7, 2.1).

We performed two additional analyses with the expert's data applying a larger spread to the prior distribution of crystalline silica exposure in the population. Firstly, we assumed logit_prev, pop _~ N(-5.30, 0.8211) which corresponds to the expert's prior as before but with an uncertainty factor of five instead of two. The posterior SMR was estimated at 1.32 with a 95% posterior interval spanning from 0.7 to 2.0. Secondly, we used a prior with an expectation equal to the CAREX prior but accompanied with a larger spread (again corresponding to a factor of 5): logit_prev, pop _~ N(-3.74, 0.8211). In this case, the posterior SMR based on expert data was estimated as 1.40, 95% posterior interval = 0.8, 2.1.

In these analyses we always used a flat prior for the SMR. We explored the robustness of this approach by applying more concentrated SMR priors. Following [9], p. 334, 336 we used alternate prior distributions for the SMR with 95% prior intervals spanning from 0.1 to 10 (corresponding to σ = log(10)/1.96 = 1.175 for log SMR) and 0.25 to 4 (corresponding to σ = log(4)/1.96 = 0.707). The standard deviations are clearly smaller than 10,000 we used in the main analyses. Based on the automatic approach (CAREX, Analysis 1) we estimated 95% posterior intervals spanning from 0.3 to 3.0 (σ = 1.175) and 0.4 to 2.6 (σ = 0.707), Analyses applying the expert data (Analysis 3) returned 95% posterior intervals of 0.7 to 2.0 (σ = 1.175 and σ = 0.707), as expected, the medians of the posterior distributions remained unchanged, i.e., they were identical to those returned by the main analyses.

Additionally we explored whether a different specification of the relative lung cancer risk of smokers/ex-smokers may affect the results considerably. We averaged (geometric mean) estimates for men (active and ex-smokers) from the Nationwide American Cancer Society prospective cohort study ([37], Table Three, full models for lung cancer) and used RR = 13.3 with 0.95-confidence limits at 11.0 and 16.0. Applying the alternate prior distribution for the SMR with 95% prior intervals spanning from 0.25 to 4 again, the analyses based on the smoking effect estimates of Thun et al. 2000 [37] returned a median posterior SMR of 1.0 with a 95% posterior interval spanning from 0.4 to 2.5 (CAREX, Analysis 1) and 1.3 (0.7, 1.9) when using expert data (Analysis 3).

Furthermore, we rerun these analyses while incorporating positive correlations between the draws of smoking prevalences among cohort and population and between the draws of silica exposure prevalences among cohort and population. It may be argued that one expects a higher prevalence among the cohort if the prevalence is higher in the population ([9], p. 371, 372). We implemented these dependencies by applying formula 19-20 in [9], p. 372, and set both correlations between the logits of prevalences to 0.8 (cp. [9], p. 374). The modified Analysis 1 (CAREX) returned a median posterior SMR of 1.0 with 95% posterior intervals spanning from 0.4 to 2.6. The results were 1.3 (0.7, 1.9) when reanalysing the expert data (Analysis 3).

**Figure 2:**
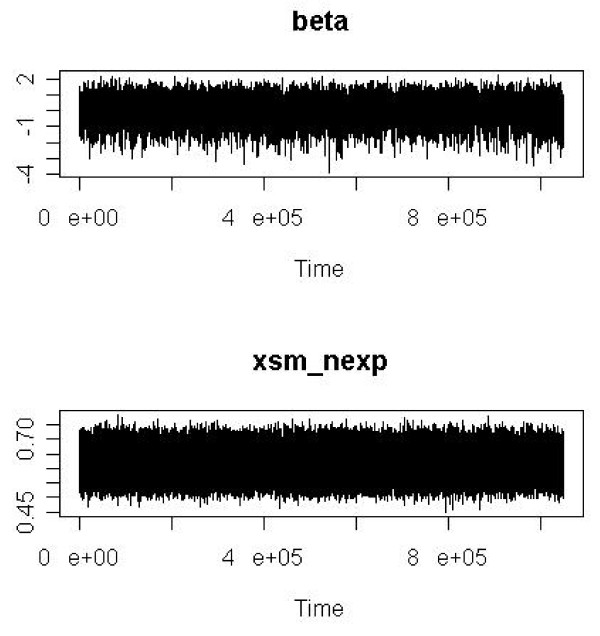
**Trace plots of log SMR (= beta) and the estimated logit of proportion of current or former smokers among unexposed to carbon black (= xsm_nexp)**. Names (beta, xsm_nexp) correspond to the variable names used in the R program (see Additional File 3). Results from an MCMC random walk of length 1,000,000 (Metropolis sampler) in Analysis 1 (CAREX, cohort smoking data). Plots include the burn-in phase of 50,000 cycles to give a complete graphical impression of the convergence behaviour of the Markov chain (Time measures 1,050,000 cycles).

**Figure 3:**
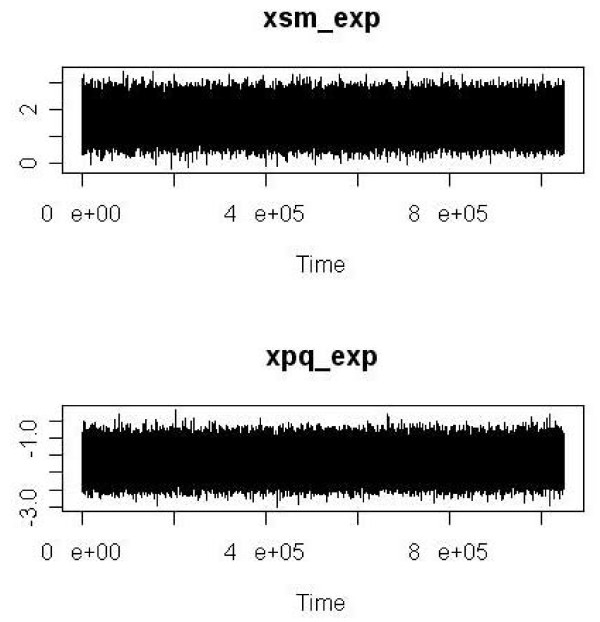
**Trace plots of logit of proportion of current or former smokers among exposed to carbon black (= xsm_exp) and logit of proportion of previously exposed to crystalline silica among exposed to carbon black (= xpq_exp)**. Names (xsm_exp, xpq_exp) correspond to the variable names used in the R program (see Additional File 3). Results from an MCMC random walk of length 1,000,000 (Metropolis sampler) in Analysis 4 (expert's assessment, case-control smoking data). Plots include the burn-in phase of 50,000 cycles to give a complete graphical impression of the convergence behaviour of the Markov chain (Time measures 1,050,000 cycles).

As simple diagnostic tools informing about goodness of sampler convergence we give trace plots for, e.g., the estimated log SMR (= beta) and the estimated logit of proportion of current or former smokers among unexposed to carbon black (= xsm_nexp) in Analysis 1 (Figure 2) and the logit of proportion of current or former smokers among exposed to carbon black (= xsm_exp) and the logit of proportion of previously exposed to crystalline silica among exposed to carbon black (= xpq_exp) in Analysis 4 (Figure 3). The names correspond to variable names as used in the R program doing the analysis (see Additional File 3). All the other estimated parameters in all four analyses showed a similar behaviour as in the examples presented in Figures 2 and 3.

## Discussion

We applied a Bayesian methodology in a cohort study of German carbon black production workers [6] to adjust the elevated lung cancer SMR of 2.18 (0.95-CI: 1.61, 2.87) for potential confounding. A nested case-control study had identified smoking and previous occupational exposures to lung carcinogens received previous to work at the carbon black plant as potential confounders [8]. We used a Markov Chain Monte Carlo approach (Metropolis sampler) to quantify the effect of the potential confounders on the SMR by calculating the distribution of the posterior SMR[32,33].

The realized acceptance rates between 20% and 40% were well in the range of published recommendations [31] and trace plots revealed no problems with the convergence behaviour of the MCMC sampler. Thus, the chosen tuning parameters and sampler length of 1,000,000 appear to be appropriate together with a burn-in phase of 50,000 cycles. Even such long Markov chains could be realized and evaluated with the R package [36] on usual laptops or PCs with run times of only a few minutes (programming code in Additional File 3).

The Bayesian analysis returned a median posterior SMR estimate in the range between 1.32 (central 0.95-region: 0.7, 2.1) and 1.00 (central 0.95-region: 0.2, 3.3) depending on how previous exposures were assessed.

The first result is based on an independent expert assessment of previous exposures combined with a conservative adjustment for smoking [5]. The second finding is based on an automatic approach (CAREX) [29,30]. The usually calculated lung cancer SMR statistic overestimated effect and precision when compared with the results from the Bayesian approach. This is particularly true when the automatic approach (CAREX) [29,30] was chosen to assess previous exposures. The difference in point estimates between both approaches resulted, at least in part, from the conservative handling of the smoking adjustment within the first approach. Additional analyses showed that the results based on the expert's assessments of prior silica dust exposure among the carbon black workers changed only slightly when the prior of the silica dust exposure distribution in the population was varied.

The CAREX system [29,30] was applied to derive estimates for crystalline silica exposure. Obviously, CAREX may give distorted estimates when applied to a specific group of workers [30]. Although the estimated level of exposure may be distorted, there is no reason to suspect a differential misclassification between cases and controls stemming from the same cohort. To validate analytical results based on CAREX estimates we used estimates of exposure probabilities generated by an independent German expert [8]. Again, we do not see a reason to believe in a differential misclassification of exposures between cases and controls. Because these approaches are very different we were not surprised getting clearly discrepant estimates of the prevalence of workers previously exposed to crystalline silica dust in the carbon black cohort: 74% (CAREX) versus 16% (expert). However, both very different approaches led to the same conclusion: the previous exposure to carcinogens received outside the carbon black plant, indicated by exposure to crystalline silica dust, clearly biased the lung cancer SMR upwards. Thus, both very different exposure estimation approaches led to similar quantitative corrections of the potentially biased SMR. This consistency is a strength and not a weakness of our Bayesian bias adjustment procedure.

These findings partially support the results from simple sensitivity analyses. A corrected lung cancer SMR was calculated as 1.33 (adjusted 0.95-CI: 0.98, 1.77) when virtually the same bias adjustments were made but with the naïve procedures as applied in our earlier analysis. The derived bias factor depended in the same degree on smoking and on previous exposures, each relative bias was estimated to be about 25%. No uncertainties of the bias parameters were taken into account in that report [5]. As expected, uncertainty was inappropriately considered in the simple analysis although the downward adjusted point estimate correctly conveyed the large impact of the two biases.

SMR analyses have often been described as prone to bias [38]. Researchers have been encouraged to consider and quantify the potential distortions or to apply alternative analytical procedures. A discussion of these described limitations of SMR analyses was given by Morfeld and co-workers [5]. The degree of adjustment derived in this study may appear surprisingly large in comparison to discussions of the impact of biases in occupational epidemiology [39]. However, appropriate simulation studies showed that a doubling of the relative risk estimate may easily be produced in realistic epidemiologic scenarios as a result of residual and unmeasured confounding [40].

Crystalline silica dust exposure is only a weak lung carcinogen [41]. Elevated lung cancer mortalities were observed [41] at cumulative exposures as high as 6 mg*m^3^-years or even higher [42] and relative risks were reported to be lower than 1.3 usually. The excess risk appears to be concentrated on people with silicosis who showed a doubled lung cancer mortality in comparison to the general population [43]. However, in our nested case-control study [8] the variable indicating previous exposure to crystalline silica dust was found to be significantly linked to lung cancer mortality with odds ratios of about 2 or 3 after adjustment for smoking and carbon black exposure. The lower estimate was based on CAREX data, the higher one on the expert's assessment. Thus, both approaches that we applied to estimate previous exposures to carcinogens resulted in clearly elevated relative risk estimates - although the previous exposure assessment approaches were independent and very different in nature. It is important to note that crystalline silica dust exposure was clearly correlated in this study with other previous exposures to carcinogens, like asbestos and PAH exposures. Thus, we interpreted the crystalline silica dust exposure variable as an indicator of exposure to a combination of carcinogens received outside the carbon black plant [8]. We did not use external relative risk data to adjust for the potential impact of previous exposures to crystalline silica dust in this study because partial data on confounders were available for the cohort of interest. Data describing the risk situation of the cohort are usually preferred in adjustment compared to external data because no additional exchangeability assumption must be accepted. It is unusual, for example, to adjust for age in a study by using population data on lung cancer age trends if an internal adjustment is possible by the age data of the cohort at hand. However, an external approach would be the only way to adjust for confounders if no data on covariate risk estimation were available for the cohort.

The latter argumentation applies also to smoking status as cause of a potential bias in occupational lung cancer epidemiology. In this bias analysis we wanted to exploit the gathered data about the workers under study to the best of our ability. However, it is important to note that additional external data about the effect of smoking (e.g., [37], as applied to a US cohort of crystalline silica exposed workers by Steenland and Greenland [12]) may help to yield narrower posterior intervals - given that these data are truly applicable to the cohort under study. A recent overview by the International Agency for Research on Cancer (IARC) [44] showed a large variation in lung cancer risk estimates between investigations (Table 2.1.1.1) and the IARC working group compiled evidence for factors affecting risk like duration and intensity of smoking, type of cigarette, type of inhalation, and population characteristics (gender, ethnicity). The smoking status variable as documented in this investigation and other epidemiological studies is only a crude measure and may also code additional life style and social class differences [45]. Thus it is not easy to judge whether externally gathered data on the smoking-lung cancer association do really apply - together with their larger precision. We hesitated to do this in the main analysis and decided to use only data in this bias adjustment that was gathered for this cohort and collected for the embedded case-control study. However, we applied additionally relative risk estimates with 0.95-confdence intervals based on the Nationwide American Cancer Society prospective cohort study [37] to explore the impact of the somewhat higher point estimate and the much smaller confidence interval on the bias correction. No substantial change in the posterior SMR estimate was observed.

The analyses presented suffer from some uncertainties not quantified. For example, our computations were based on the assumption that the odds ratios from the nested case-control study analyses estimated the relative risks for the cohort in a suitable way and that the proportion of subjects with previous occupational exposure to crystalline silica was representative for the cohort. Such an assumption may hold for the smoking distribution although based on the sub-cohort with smoking information only but can be questioned for cumulative carbon black exposure [8]. Thus, the question remains whether the controls were representative for the previous exposure distribution among the cohort members. Moreover, some distortions may be due to a small sample bias [46], an argument relevant for the analyses based on the expert assessment of the data. We applied a conservative correction for smoking, as noted in an earlier paper, to reduce this potential bias [8].

We applied crude estimates ("guessed factors") to quantify the instability of CAREX percentages of occupational crystalline silica exposure in the population and used an additional adaptation factor to derive population prevalence estimates for an analysis applying expert assessment data. Although these factors could have been varied in additional sensitivity analyses, we believe that the uncertainty of these "guesses" is of minor impact on the results because posterior SMRs showed a wide spread and the performed sensitivity investigation on the expert population prior returned no relevant impact of the varied prior parameter values on the result.

Whether the derived prior distributions are independent from one another is worthy of discussion. External exposures and tobacco consumption showed some positive correlation [8], however, correction factors for previous exposures were derived after adjustment for smoking. We therefore believe that the correction of the SMR distortion attributed to previous exposures is independent of the bias correction due to the differing smoking behaviour in the cohort and the population. A further assumption made is that the confounding biases are independent of the confounding from measured variables (like age and calendar time). However, our ability to specify such correlations knowledgeably is clearly limited and almost all bias analyses rely on this assumption (e.g., [12,13,47], see chapter 19 of [9]). However, implementing correlations of 0.8 between the draws of the logit of smoking prevalences among cohort and population and between the draws of the logit of crystalline silica prevalences among cohort and population did change the results only negligibly.

A possible distortion due to a healthy worker survivor bias cannot be ruled out. However, in Cox analyses of this cohort it was tried to separate out past aspects of exposure to adjust for survivor biases [7]. This approach is not fully appropriate. A far better and more valid adjustment can be performed by G-estimation [48-51]. However, a considerable amount of detailed longitudinal data is necessary to generate enough power if that procedure is applied [52]. Due to the severe power restriction in this study (only 50 lung cancer deaths available) an application of Robins' G-estimation will be to no avail. Thus, distortions due to possible survivor selection effects are not satisfactorily resolved.

We followed Steenland and Greenland [12] and applied like de Vocht and co-workers [13] an uninformative prior for the SMR. Strictly speaking, such priors are strange because extremely high and extremely low relative risks are given the same probability as relative risks in a "normal" range between 0.25 and 4 [18,53]. Thus, it is indicated to apply an informative prior that is more realistic [9,54]. However, the prior should be "broad enough to assign relatively high probability to each discussant's opinion" [15]. A possible occupational cancer risk due to carbon black exposure is indicated by the occurrence of lung cancer in rats after inhalation or instillation of carbon black and, thus, a working group at the International Agency for Research on Cancer concluded that there is sufficient evidence in experimental animals for the carcinogenicity of carbon black and carbon black extracts [4]. However, Valberg and co-workers [55] summarized their overview on the carcinogenicity of carbon black as follows: "new epidemiological evidence decreases concerns for cancer risks compared with the pre-1996 evidence. Laboratory studies support a conclusion that the mechanism of tumorigenicity of CB [carbon black] in rats is no different from that of any poorly soluble particles, ie, toxicity results from the particle overload per se, and not from the particles' chemistry". A leading working group in particle toxicology concluded [56] that the observed carcinogenic effect of carbon black is only specific for rats and that the effects cannot be demonstrated in mice or hamsters. Moreover, they proposed a threshold effect in rats implying that no cancer hazard exists in rats (and humans) if exposures are controlled accordingly. This judgement of a limited relevance of the positive rat experiments with poorly soluble particles is in line with the consensus report of an expert workshop held at the ILSI institute [57]. Thus, we think it appropriate to apply a flat prior to the SMR in this analysis covering without doubt all these opinions about the potential carcinogenicity of carbon black. Like Steenland and Greenland [12] we do not think that is a major drawback to include absurd large and small priors also. The applied flat SMR prior distribution may help to convince scientists from other faculties not acquainted with Bayesian thinking that such a bias analysis is a worthwhile exercise - or even may convince frequentists who resist to a non-flat prior distribution of the target parameter [58].

However, we explored the robustness of our findings by applying more concentrated SMR priors. Alternate prior distributions for the SMR with 95% prior intervals spanning from 0.1 to 10 and 0.25 to 4 produced somewhat narrower interval estimates. The main effect was a lowering of the upper limits in the CAREX based analyses. These results did not indicate that the main analyses based on flat priors for the SMR were misleading.

Our finding that the elevated lung cancer SMR in the German study cannot be taken as proof for a causal impact of carbon black exposure on lung cancer risk is consistent with the recent decision of an IARC working group to classify carbon black - based on positive findings in rat experiments - as Group 2B ("possibly carcinogenic") but not as a human lung carcinogen [4]. The working group stated that "there is *inadequate evidence *in humans for the carcinogenicity of carbon black" [4]. Further support of this decision was given by an updated analysis of two large case-control studies in Montreal: "Subjects with occupational exposure to carbon black... did not experience any detectable excess risk of lung cancer" [59].

Bayesian bias analyses - and to a lesser extent, as an approximation, Monte Carlo sensitivity analyses - are recommended for combining background data about biases from different sources [12] p.385, [60] p.47, [9] p.378-380. In contrast to Monte Carlo Sensitivity Analyses the Bayesian method follows a clear mathematical and philosophical rationale [10], [60] p.53; chapter 18 of [9], [17]. The Bayesian approach can take into account correlations between multiple bias estimates and their different precisions [11]. Even crude estimates of the probable degree of distortion can be included. This method is especially valuable when observational studies are performed with a large number of subjects (high precision) to quantify small effects sizes (low risk) as often in genetic or environmental epidemiology [61]. Bayesian analyses may also help to cope with inflated associations [61]. Overstated effect estimates are to be expected even if the study is unbiased in the usual sense [62]. Other authors have also noted the value of Bayesian analyses, e.g., to reduce false positives in epidemiology [63]. Bayesian false discovery probability is also currently used in the analysis of large genetic data bases where the danger is rather large that conventional analytical analyses label spurious associations as noteworthy [64]. Another important application is smoothing by hierarchical Bayesian models [65]. Markov Chain Monte Carlo Methods (MCMC) can be used in these analyses and to perform a Bayesian bias correction, the objective of this paper, in simple and complicated scenarios [31]. Programming can be done with standard software packages like the R program [36]. Other recent applications of Bayesian methods for correcting unmeasured confounding [13] and misclassification [66] in epidemiological studies are promising examples that this analytical technique may become a common tool in epidemiology. Thus, Bayesian bias adjustment can become a valuable adjunct in occupational and environmental epidemiology to overcome narrative discussions of potential distortions.

## Conclusions

Bayesian bias adjustment is an excellent tool to quantitatively combine data about confounders from different sources. Markov Chain Monte Carlo Methods (MCMC) can be used to evaluate Bayes' theorem even in complicated scenarios. Programming can be done with standard software like R that is readily available on the web. Thus, Bayesian bias adjustment can become a regular tool in occupational and environmental epidemiology to overcome narrative discussions of potential distortions.

We studied a statistically elevated lung cancer SMR of 2.18 (0.95-CI: 1.61, 2.87) in a German carbon black production worker cohort with Bayesian techniques. No link with carbon black exposure in internal analyses was noted; potential confounders such as smoking and previous occupational exposures to carcinogens identified by a nested case-control study showed that the normally calculated lung cancer SMR overestimated effect and precision when compared with the MCMC results [median posterior SMR estimate in the range between 1.32 (central 0.95-region: 0.7, 2.1) and 1.00 (central 0.95-region: 0.2, 3.3) depending on the method how previous exposures were assessed]. This finding is consistent with the conclusion of an IARC working group in 2006 not to classify carbon black as a human lung carcinogen.

## Competing interests

This study was supported by a grant from the International Carbon Black Association (ICBA). Both authors serve as Scientific Advisors to this Association. However, the authors declare that they do not have a conflict of interest. The ICBA http://www.carbon-black.org is a scientific, non-profit corporation originally founded in 1977. The purpose of the ICBA is to sponsor, conduct, and participate in investigations, research, and analyses relating to the health, safety, and environmental aspects of the production and use of carbon black. The manuscript was neither influenced by the ICBA nor by any company funding the ICBA nor does it present any view or opinion of the ICBA or of the companies.

## Authors' contributions

PM developed the methodology, programmed the code and performed the analysis. RJM discussed the occupational background (carbon black production and exposures). Both authors read and approved the final manuscript.

## Supplementary Material

Additional file 1 **Glossary of key terms**. Key terms of the Bayesian analysis and its implementation are explained. Click here for file

Additional file 2 **Derivation of the bias factor**. The bias factor equation is explained in detail which is applied throughout in the analyses. Click here for file

Additional file 3 **R program code**. R program for Bayesian bias adjustment of a potentially distorted SMR via Markov Chain Monte Carlo simulation (Metropolis sampler). Click here for file
