# Supplementary material for: Bayesian bias adjustments of the lung cancer SMR in a cohort of German carbon black production workers
Source: J Occup Med Toxicol. 2010 Aug 11;5:23. doi: 10.1186/1745-6673-5-23 (PMC2928247; doi:10.1186/1745-6673-5-23)
Supplement: Additional file 1 — Glossary of key terms. Key terms of the Bayesian analysis and its implementation are explained. [file 1745-6673-5-23-S1.DOC]

**Additional File 1**

**Glossary of key terms**

Potentially Biased SMR: The SMR, i.e., the usual mortality ratio standardized for gender, age and calendar time is potentially biased by confounders, like smoking, that are ignored in the standardization process. Thus, the usual SMR is potentially biased due to confounders different from age, gender and calendar time.

Posterior SMR: The SMR adjusted by a Bayesian bias adjustment procedure. The Bayesian procedure can apply information about partially measured or even unmeasured confounders to adjust the potentially biased SMR accordingly.

Prior (prior distribution): Information about the parameter of interest, like the SMR, and its potential distortions that is prior (outside) to the data of the study. Because the prior is outside the study it cannot be incorporated into a standard analysis like data on confounders measured on subjects enrolled for study.

Likelihood of data: probability of the observed data in a study given a statistical model that is assumed to generate the data. Core term of frequentist statistics (standard statistics) also used in Bayesian statistics.

Markov Chain Monte Carlo (MCMC): The MCMC approach embraces a class of statistical techniques to generate a sample from a (complicated) joint probability distribution. One algorithm in this class of techniques is the Metropolis algorithm. This algorithm can be used to approximate the posterior distribution in a Bayesian analysis.
