# Supplementary material for: Bayesian bias adjustments of the lung cancer SMR in a cohort of German carbon black production workers
Source: J Occup Med Toxicol. 2010 Aug 11;5:23. doi: 10.1186/1745-6673-5-23 (PMC2928247; doi:10.1186/1745-6673-5-23)
Supplement: Additional file 2 — Derivation of the bias factor. The bias factor equation is explained in detail which is applied throughout in the analyses. [file 1745-6673-5-23-S2.DOC]

**Additional File 2**

**Bias factor**

Following ideas developed and applied by Cornfield et al. 1959 [21] (reprinted as Cornfield et al. 2009 [22]), Bross 1966 [23], Yanagawa 1984 [24] or Axelson and Steenland 1988 [25] we derive a bias factor that estimates the distortion of the SMR due to uncontrolled confounding by smoking. Let RR denote the observed relative risk, cohort to population (= SMR); Rij the true risk with i = employed at carbon black plant (0 = no, 1= yes), j = smoker or ex-smoker (0 = no, yes = 1); prop_smoke,coh_ : proportion of smokers/ex-smokers in the carbon black cohort; prop_smoke,pop_ : proportion of smokers/ex-smokers in the general population; OR_smoke_: odds ratio for smokers/ex-smokers vs. never smokers. Then

RR_corr_ = R_10_ / R_00_ is the true (counterfactual) relative risk we would have observed had all subjects of the cohort and all subjects of the population been never-smokers. The bias factor bias_smoke_ is given by the equation RR = bias_smoke_ * RR_corr_. Therefore, with RR_smoke_ = OR_smoke_, we get for the bias factor

Of course, this derivation assumes that the adjuster applied forms a sufficient set of control.[9, 10].
