# Supplementary material for: Bayesian bias adjustments of the lung cancer SMR in a cohort of German carbon black production workers
Source: J Occup Med Toxicol. 2010 Aug 11;5:23. doi: 10.1186/1745-6673-5-23 (PMC2928247; doi:10.1186/1745-6673-5-23)
Supplement: Additional file 3 — R program code. R program for Bayesian bias adjustment of a potentially distorted SMR via Markov Chain Monte Carlo simulation (Metropolis sampler). [file 1745-6673-5-23-S3.DOC]

**Additional File 3**

**R program for Bayesian bias adjustment of a potentially distorted SMR
via Markov Chain Monte Carlo simulation (Metropolis sampler)**

# BAYESIAN BIAS ANALYSIS: MCC WITH METROPOLIS GENERATOR

# Analysis 1 acc to Table 1 in Morfeld and McCunney 2010

sink("J:/Morfeld/R_work/analysis_1.txt") # output file

d=date()

cat("\nTime:",d)

set.seed(882) # seed of random number generator

cat("\nset.seed(882): all results are reproducible!\n\n")

cat("\nAnalysis 1: \n\n")

cat("\nBAYESIAN BIAS ANALYSIS (METROPOLIS GENERATOR)\n")

cat("\nMorfeld et al. : Lung cancer mortality and carbon black exposure -")

cat("\nUncertainties of SMR analyses in a cohort study at a German carbon black production plant.")

cat("\nJOEM 2006; 48, 1253-1264\n\n")

cat("\ncp. Steenland and Greenland: Monte Carlo Sensitivity Analysis...")

cat ("\tAm J Epidemiol 2004;160:384-392")

cat("\nKen Newman, Bayesian Inference (Lecture Notes), Spring 2005")

cat("\thttp://www.creem.st-and.ac.uk/ken/\n\n")

# ****************************************************************************************************

cat("\n\nPRIOR PARAMETERS (n=7):

\t beta.star beta = log SMR

\t xsm_exp.star logit of proportion of current or former smokers among exposed (to carbon black)

\t xsm_nexp.star logit of proportion of current or former smokers among unexposed (to carbon black)

\t xpq_exp.star logit of proportion of prior exposed (to silica) among exposed (to carbon black)

\t xpq_nexp.star logit of proportion of prior exposed (to silica) among unexposed (to carbon black)

\t bsm.star log rate ratio of smokers

\t bpq.star log rate ratio of prior silica exposure\n\n")

# ****************************************************************************************************

# this evaluates the LIKELIHOOD FUNCTION

like.fun=function(beta_prior,xsm_exp,xpq_exp,xsm_nexp,xpq_nexp,bsm,bpq) {

psm1=exp(xsm_exp)/(1+exp(xsm_exp))

# proportion of smokers among exposed (to carbon black)

psm0=exp(xsm_nexp)/(1+exp(xsm_nexp))

# proportion of never smokers among unexposed (to carbon black), derived from German survey

pns1=1-psm1

# proportion of never smokers among exposed (to carbon black)

pns0=1-psm0

# proportion of never smokers among unexposed (to carbon black), derived from German survey

bias_sm=(pns1+exp(bsm)*(psm1))/(pns0+exp(bsm)*(psm0))

# smoking: bias parameter

ppq1=exp(xpq_exp)/(1+exp(xpq_exp))

# proportion with previous exposure (to silica) among exposed (to carbon black)

pnpq1=1-ppq1

# proportion without previous exposure (to silica) among exposed (to carbon black)

ppq0=exp(xpq_nexp)/(1+exp(xpq_nexp))

# proportion with previous exposure (to silica) among unexposed (to carbon black)

pnpq0=1-ppq0

# proportion without previous exposure (to silica). among unexposed (to carbon black)

bias_pq=(pnpq1+exp(bpq)*(ppq1))/(pnpq0+exp(bpq)*(ppq0))

# previous exposure to silica: bias parameter

bias=bias_sm*bias_pq

# combined bias

beta_obs=beta_prior+log(bias)

# log SMR observed (confounded) = prior log SMR (unconfounded) + log(bias)

lambda=22.94*exp(beta_obs)

# observed number of deaths are distributed as a Poisson variable with mean lambda=expected*SMR

# SMR=2.18, 50/2.18=22.94

likelihood=dpois(50,lambda)

# one observation only

return(likelihood)

}

# ****************************************************************************************************

# METROPOLIS SAMPLER INITIATION

Nb=50000 # burn-in phase

N=1000000 # evaluation phase

cat("\n\nBURN-IN AND LENGTH OF FINAL CHAIN:

\t length of burn-in chain=",Nb,

"\n\t length of generating chain=",N,"\n\n")

# Acceptance rate should be between 15 % und 50 % acc to Roberts 1996, p. 55

# Roberts G. (1996): "Markov chain concepts related to sampling algorithms"

# in Gilks, Richardson and Spiegelhalter eds, Markov Chain Monte Carlo in Practice, pp 45-57

# sigmas as tuning parameters of MCMC :

sigma.beta=0.5 # the tuning parameter for the proposal for beta

sigma.xsm_exp=0.3 # the tuning parameter for the proposal for xsm_exp

sigma.xpq_exp=1 # the tuning parameter for the proposal for xpq_exp

sigma.xsm_nexp=0.1 # the tuning parameter for the proposal for xsm_nexp

sigma.xpq_nexp=1 # the tuning parameter for the proposal for xpq_nexp

sigma.bsm=3 # the tuning parameter for the proposal for bsm

sigma.bpq=1 # the tuning parameter for the proposal for bpq

cat("\n\nTUNING PARAMETERS:

\t sigma.beta=",sigma.beta,

"\n\t sigma.xsm_exp=",sigma.xsm_exp,

"\n\t sigma.xpq_exp=",sigma.xpq_exp,

"\n\t sigma.xsm_nexp=",sigma.xsm_nexp,

"\n\t sigma.xpq_nexp=",sigma.xpq_nexp,

"\n\t sigma.bsm=",sigma.bsm,

"\n\t sigma.bpq=",sigma.bpq,"\n\n")

U.beta=runif(Nb+N) # uniformly distributed variable to evaluate the Metrop-Hast-Fraction for beta

U.xsm_exp=runif(Nb+N) # uniformly distributed variable to evaluate the Metrop-Hast-Fraction for xsm_exp

U.xsm_nexp=runif(Nb+N) # uniformly distributed variable to evaluate the Metrop-Hast-Fraction for xsm_nexp

U.xpq_exp=runif(Nb+N) # uniformly distributed variable to evaluate the Metrop-Hast-Fraction for xpq_exp

U.xpq_nexp=runif(Nb+N) # uniformly distributed variable to evaluate the Metrop-Hast-Fraction for xpq_nexp

U.bsm=runif(Nb+N) # uniformly distributed variable to evaluate the Metrop-Hast-Fraction for bsm

U.bpq=runif(Nb+N) # uniformly distributed variable to evaluate the Metrop-Hast-Fraction for bpq

accept.beta=0 # the number accepted for beta

accept.xsm_exp=0 # the number accepted for xsm_exp

accept.xsm_nexp=0 # the number accepted for xsm_nexp

accept.xpq_exp=0 # the number accepted for xpq_exp

accept.xpq_nexp=0 # the number accepted for xpq_nexp

accept.bsm=0 # the number accepted for bsm

accept.bpq=0 # the number accepted for bpq

nan.beta=0 # the number of nan's for Metrop-Hast-Fraction for beta

nan.xsm_exp=0 # the number of nan's for Metrop-Hast-Fraction for xsm_exp

nan.xsm_nexp=0 # the number of nan's for Metrop-Hast-Fraction for xsm_nexp

nan.xpq_exp=0 # the number of nan's for Metrop-Hast-Fraction for xpq_exp

nan.xpq_nexp=0 # the number of nan's for Metrop-Hast-Fraction for xpq_nexp

nan.bsm=0 # the number of nan's for Metrop-Hast-Fraction for bsm

nan.bpq=0 # the number of nan's for Metrop-Hast-Fraction for bpq

beta.star=rep(NA,Nb+N) # space for the beta sample values (log SMR)

xsm_exp.star=rep(NA,Nb+N) # space for the xsm_exp sample values (logit of proportion of smokers, exposed)

xsm_nexp.star=rep(NA,Nb+N) # space for the xsm_nexp sample values (logit of proportion of smokers, unexposed)

xpq_exp.star=rep(NA,Nb+N) # space for the xpq_exp sample values (logit of proportion of silica, exposed)

xpq_nexp.star=rep(NA,Nb+N) # space for the xpq_nexp sample values (logit of proportion of silica, unexposed

bsm.star=rep(NA,Nb+N) # space for the bsm sample values (log lung cancer rate ratio for smokers)

bpq.star=rep(NA,Nb+N) # space for the bpq sample values (log lung cancer rate ratio for silica)

beta.star[1]=0 # initial value: log SMR=0, i.e., SMR=1

# flat prior distribution: log SMR distr as N(0;10^8)

xsm_exp.star[1]=1.66 # initial value: mean logit of proportion of smokers, exposed (logit 84%)

# cohort with smoking info, 1180 workers: s(xsm_exp) = (1180*0.84*0.16)^(-1/2) = 0.0794

xpq_exp.star[1]=1.05 # initial value: mean logit of proportion of prior exposed (to silica), exposed (logit 74%)

# NCC, 88 workers (control subjects): s(xsm-exp) = (88*0.74*0.26)^(-1/2) = 0.243

xsm_nexp.star[1]=0.62 # initial value: mean logit of proportion of current smokers, unexposed (logit 65%)

# Nagel, Junge and Bundesgesundheitssurvey 1998:

# 3450 men, i.e., logit(0.65) = 0.619, s(xsm_nexp) = (3450*0.65*0.35)^(-1/2) = 0.0357

xpq_nexp.star[1]=-3.7480324 # initial value: mean logit of proportion of prior exposed (to silica), unexposed (logit 2.3%)

# proposed 0.95-CI: 1.1373554%, 4.6043165% =2*mean,

# i.e., std dev of logit silica acc to Carex: .36581292

bsm.star[1]=2.23 # initial value: mean log lung cancer rate ratio for smokers,

# Buechte et al. 2006 (on-line section): OR=9.27; 0.95-CI=1.16, 74.4;
 # i.e., lnOR=2.227, s(bsm) = ln(74.4/1.16)/3.92 = 1.061

bpq.star[1]=0.74 # initial value: mean log lung cancer rate ratio for prior exposed (to silica),

# Morfeld et al. 2006, Table 7: OR=2.04

# Buechte et al. 2006, p. 1274: OR=2.1; 0.95-CI= 0.39, 11.2; i.e.,

# lnOR = ln(2.1) = 0.74, s(bpq) = ln(11.2/0.39)/3.92 = 0.8565

cat("\n\INITIAL VALUES:

\t beta.star[1]=",beta.star[1],

"\n\t xsm_exp.star[1]=",xsm_exp.star[1],

"\n\t xpq_exp.star[1]=",xpq_exp.star[1],

"\n\t xsm_nexp.star[1]=",xsm_nexp.star[1],

"\n\t xpq_nexp.star[1]=",xpq_nexp.star[1],

"\n\t bsm.star[1]=",bsm.star[1],

"\n\t bpq.star[1]=",bpq.star[1],"\n\n")

# ****************************************************************************************************

# METROPOLIS SAMPLER with Gaussian random walk generator for candidate values

for (r in 2:(Nb+N)) {

# BETA

# initial value: log SMR=0, i.e., SMR=1

# flat prior distribution: log SMR distr as N(0;10^8)

beta.can=rnorm(1,beta.star[r-1],sigma.beta) # generating BETA candidate

fraction=(like.fun(beta.can,xsm_exp.star[r-1],xpq_exp.star[r-1],xsm_nexp.star[r-1],xpq_nexp.star[r-1],bsm.star[r-1],bpq.star[r-1])

*dnorm(beta.can,0,10000))/

(like.fun(beta.star[r-1],xsm_exp.star[r-1],xpq_exp.star[r-1],xsm_nexp.star[r-1],xpq_nexp.star[r-1],bsm.star[r-1],bpq.star[r-1])

*dnorm(beta.star[r-1],0,10000))

# ratio of posterior densities (product of poisson likelihood and flat Gaussian prior)

# at beta.can and beta.star[r-1] using current estimates of other parameters

metro.hastings.frac=min(1,fraction)

if (is.na(metro.hastings.frac)) {

metro.hastings.frac=0.5 # at hoc when fraction does not exist

nan.beta=nan.beta+1 # number of not-existing fractions

}

if(U.beta[r]<metro.hastings.frac) {

beta.star[r]=beta.can

accept.beta=accept.beta+1

} else

{ beta.star[r]=beta.star[r-1]

}

# XSM_EXP

# initial value: mean logit of proportion of smokers, exposed (logit 84%=1.66)

# cohort with smoking info, 1180 workers: s(xsm_exp) = (1180*0.84*0.16)^(-1/2) = 0.0794

xsm_exp.can=rnorm(1,xsm_exp.star[r-1],sigma.xsm_exp) # generating XSM_EXP candidate

fraction=(like.fun(beta.star[r],xsm_exp.can,xpq_exp.star[r-1],xsm_nexp.star[r-1],xpq_nexp.star[r-1],bsm.star[r-1],bpq.star[r-1])

*dnorm(xsm_exp.can,1.66,0.0794))/

(like.fun(beta.star[r],xsm_exp.star[r-1],xpq_exp.star[r-1],xsm_nexp.star[r-1],xpq_nexp.star[r-1],bsm.star[r-1],bpq.star[r-1])

*dnorm(xsm_exp.star[r-1],1.66,0.0794))

# ratio of posterior densities (product of poisson likelihood and Gaussian prior)

# at xsm_exp.can and xsm_exp.star[r-1] using current estimates of other parameters

metro.hastings.frac=min(1,fraction)

if (is.na(metro.hastings.frac)) {

metro.hastings.frac=0.5 # at hoc when fraction does not exist

nan.xsm_exp=nan.xsm_exp+1 # number of not-existing fractions

}

if(U.xsm_exp[r]<metro.hastings.frac) {

xsm_exp.star[r]=xsm_exp.can

accept.xsm_exp=accept.xsm_exp+1

} else

{ xsm_exp.star[r]=xsm_exp.star[r-1]

}

# XPQ_EXP

# initial value: mean logit of proportion of prior exposed (to silica), exposed (logit 74%=1.05)

# NCC, 88 workers (control subjects): s(xsm-exp) = (88*0.74*0.26)^(-1/2) = 0.243

xpq_exp.can=rnorm(1,xpq_exp.star[r-1],sigma.xpq_exp) # generating XPQ_EXP candidate

fraction=(like.fun(beta.star[r],xsm_exp.star[r],xpq_exp.can,xsm_nexp.star[r-1],xpq_nexp.star[r-1],bsm.star[r-1],bpq.star[r-1])

*dnorm(xpq_exp.can,1.05,0.243))/

(like.fun(beta.star[r],xsm_exp.star[r],xpq_exp.star[r-1],xsm_nexp.star[r-1],xpq_nexp.star[r-1],bsm.star[r-1],bpq.star[r-1])

*dnorm(xpq_exp.star[r-1],1.05,0.243))

# ratio of posterior densities (product of poisson likelihood and Gaussian prior)

# at xpq_exp.can and xpq_exp.star[r-1] using current estimates of other parameters

metro.hastings.frac=min(1,fraction)

if (is.na(metro.hastings.frac)) {

metro.hastings.frac=0.5 # at hoc when fraction does not exist

nan.xpq_exp=nan.xpq_exp+1 # number of not-existing fractions

}

if(U.xpq_exp[r]<metro.hastings.frac) {

xpq_exp.star[r]=xpq_exp.can

accept.xpq_exp=accept.xpq_exp+1

} else

{ xpq_exp.star[r]=xpq_exp.star[r-1]

}

# XSM_NEXP

# initial value: mean logit of proportion of current smokers, unexposed (logit 65%=0.62)

# Nagel, Junge and Bundesgesundheitssurvey 1998:

# 3450 men, i.e., logit(0.65) = 0.619, s(xsm_nexp) = (3450*0.65*0.35)^(-1/2) = 0.0357

xsm_nexp.can=rnorm(1,xsm_nexp.star[r-1],sigma.xsm_nexp) # generating XSM_NEXP candidate

fraction=(like.fun(beta.star[r],xsm_exp.star[r],xpq_exp.star[r],xsm_nexp.can,xpq_nexp.star[r-1],bsm.star[r-1],bpq.star[r-1])

*dnorm(xsm_nexp.can,0.62,0.0357))/

(like.fun(beta.star[r],xsm_exp.star[r],xpq_exp.star[r],xsm_nexp.star[r-1],xpq_nexp.star[r-1],bsm.star[r-1],bpq.star[r-1])

*dnorm(xsm_nexp.star[r-1],0.62,0.0357))

# ratio of posterior densities (product of poisson likelihood and Gaussian prior)

# at xsm_nexp.can and xsm_nexp.star[r-1] using current estimates of other parameters

metro.hastings.frac=min(1,fraction)

if (is.na(metro.hastings.frac)) {

metro.hastings.frac=0.5 # at hoc when fraction does not exist

nan.xsm_nexp=nan.xsm_nexp+1 # number of not-existing fractions

}

if(U.xsm_nexp[r]<metro.hastings.frac) {

xsm_nexp.star[r]=xsm_nexp.can

accept.xsm_nexp=accept.xsm_nexp+1

} else

{ xsm_nexp.star[r]=xsm_nexp.star[r-1]

}

# XPQ_NEXP

# initial value: mean logit of proportion of prior exposed (to silica), unexposed (logit 2.3%]=-3.7480324)

# proposed 0.95-CI: 1.1373554%, 4.6043165% =2*mean,

# i.e., std dev of logit silica acc to Carex: .36581292

xpq_nexp.can=rnorm(1,xpq_nexp.star[r-1],sigma.xpq_nexp) # generating XPQ_NEXP candidate

fraction=(like.fun(beta.star[r],xsm_exp.star[r],xpq_exp.can,xsm_nexp.star[r],xpq_nexp.can,bsm.star[r-1],bpq.star[r-1])

*dnorm(xpq_nexp.can,-3.7480324,.36581292))/

(like.fun(beta.star[r],xsm_exp.star[r],xpq_exp.star[r],xsm_nexp.star[r],xpq_nexp.star[r-1],bsm.star[r-1],bpq.star[r-1])

*dnorm(xpq_nexp.star[r-1],-3.7480324,.36581292))

# ratio of posterior densities (product of poisson likelihood and Gaussian prior)

# at xpq_nexp.can and xpq_nexp.star[r-1] using current estimates of other parameters

metro.hastings.frac=min(1,fraction)

if (is.na(metro.hastings.frac)) {

metro.hastings.frac=0.5 # at hoc when fraction does not exist

nan.xpq_nexp=nan.xpq_nexp+1 # number of not-existing fractions

}

if(U.xpq_nexp[r]<metro.hastings.frac) {

xpq_nexp.star[r]=xpq_nexp.can

accept.xpq_nexp=accept.xpq_nexp+1

} else

{ xpq_nexp.star[r]=xpq_nexp.star[r-1]

}

# BSM

# initial value: mean log lung cancer rate ratio for smokers,

# Buechte et al. 2006 (on-line section): OR=9.27; 0.95-CI=1.16, 74.4;
# i.e., lnOR=2.227, s(bsm) = ln(74.4/1.16)/3.92 = 1.061

bsm.can=rnorm(1,bsm.star[r-1],sigma.bsm) # generating BSM candidate

fraction=(like.fun(beta.star[r],xsm_exp.star[r],xpq_exp.star[r],xsm_nexp.star[r],xpq_nexp.star[r],bsm.can,bpq.star[r-1])

*dnorm(bsm.can,2.23,1.061))/

(like.fun(beta.star[r],xsm_exp.star[r],xpq_exp.star[r],xsm_nexp.star[r],xpq_nexp.star[r],bsm.star[r-1],bpq.star[r-1])

*dnorm(bsm.star[r-1],2.23,1.061))

# ratio of posterior densities (product of poisson likelihood and Gaussian prior)

# at bsm.can and bsm.star[r-1] using current estimates of other parameters

metro.hastings.frac=min(1,fraction)

if (is.na(metro.hastings.frac)) {

metro.hastings.frac=0.5 # at hoc when fraction does not exist

nan.bsm=nan.bsm+1 # number of not-existing fractions

}

if(U.bsm[r]<metro.hastings.frac) {

bsm.star[r]=bsm.can

accept.bsm=accept.bsm+1

} else

{ bsm.star[r]=bsm.star[r-1]

}

# BPQ

# initial value: mean log lung cancer rate ratio for prior exposed (to silica),

# Morfeld et al. 2006, Table 7: OR=2.04

# Buechte et al. 2006, p. 1274: OR=2.1; 0.95-CI= 0.39, 11.2; i.e.,

# lnOR = ln(2.1) = 0.74, s(bpq) = ln(11.2/0.39)/3.92 = 0.8565

bpq.can=rnorm(1,bpq.star[r-1],sigma.bpq) # generating BPQ candidate

fraction=(like.fun(beta.star[r],xsm_exp.star[r],xpq_exp.star[r],xsm_nexp.star[r],xpq_nexp.star[r],bsm.star[r],bpq.can)

*dnorm(bpq.can,0.74,0.8565))/

(like.fun(beta.star[r],xsm_exp.star[r],xpq_exp.star[r],xsm_nexp.star[r],xpq_nexp.star[r],bsm.star[r],bpq.star[r-1])

*dnorm(bpq.star[r-1],0.74,0.8565))

# ratio of posterior densities (product of poisson likelihood and Gaussian prior)

# at bpq.can and bpq.star[r-1] using current estimates of other parameters

metro.hastings.frac=min(1,fraction)

if (is.na(metro.hastings.frac)) {

metro.hastings.frac=0.5 # at hoc when fraction does not exist

nan.bpq=nan.bpq+1 # number of not-existing fractions

}

if(U.bpq[r]<metro.hastings.frac) {

bpq.star[r]=bpq.can

accept.bpq=accept.bpq+1

} else

{ bpq.star[r]=bpq.star[r-1]

}

}

# ****************************************************************************************************

# OUTPUT

cat("\n\n*** RESULTS OF BAYESIAN ANALYSIS (METROPOLIS SAMPLER) ***\n")

# TRACING

theta.out=matrix(cbind(beta.star),Nb+N,1)

dimnames(theta.out)=list(NULL,c("beta"))

# Trace plots

theta.out=matrix(cbind(beta.star,xsm_nexp.star),Nb+N,2)

dimnames(theta.out)=list(NULL,c("beta","xsm_nexp"))

# Trace plots

jpeg(file="J:/Morfeld/R_work/Metropolis_carbon_black_smoking_prior-exposure_trace_plots_beta_xsm_nexp_Analysis_1.jpeg")

par(mfrow=c(2,1))

ts.plot(as.ts(theta.out[,1]),main="beta")

ts.plot(as.ts(theta.out[,2]),main="xsm_nexp")

dev.off()

theta.out=matrix(cbind(xsm_exp.star,xpq_exp.star),Nb+N,2)

dimnames(theta.out)=list(NULL,c("xsm_exp","xpq_exp"))

# Trace plots

jpeg(file="J:/Morfeld/R_work/Metropolis_carbon_black_smoking_prior-exposure_trace_plots_xsm_exp_xpq_exp_Analysis_1.jpeg")

par(mfrow=c(2,1))

ts.plot(as.ts(theta.out[,1]),main="xsm_exp")

ts.plot(as.ts(theta.out[,2]),main="xpq_exp")

dev.off()

theta.out=matrix(cbind(bsm.star,bpq.star),Nb+N,2)

dimnames(theta.out)=list(NULL,c("bsm","bpq"))

# Trace plots

jpeg(file="J:/Morfeld/R_work/Metropolis_carbon_black_smoking_prior-exposure_trace_plots_bsm_bpq_Analysis_1.jpeg")

par(mfrow=c(2,1))

ts.plot(as.ts(theta.out[,1]),main="bsm")

ts.plot(as.ts(theta.out[,2]),main="bpq")

dev.off()

theta.out=matrix(cbind(xsm_exp.star,xpq_nexp.star),Nb+N,2)

dimnames(theta.out)=list(NULL,c("xsm_exp","xpq_nexp"))

# Trace plots

jpeg(file="J:/Morfeld/R_work/Metropolis_carbon_black_smoking_prior-exposure_trace_plots_xsm_exp_xpq_nexp_A.jpeg")

par(mfrow=c(2,1)) # die folgenden plots auf eine Seite (2 Reihen, 1 Spalte), erst Reihen füllen

ts.plot(as.ts(theta.out[,1]),main="xsm_exp")

ts.plot(as.ts(theta.out[,2]),main="xpq_nexp")

dev.off()

# SUMMARY STATISTICS FROM POSTERIOR

burn=1:Nb

cat("\n\nfinal Gaussian random walk (without burn-in):")

print(length(beta.star[-burn]))

cat("\nposterior BETA (without burn-in)\n")

print(summary(beta.star[-burn]))

cat(" standard deviation:")

print(sd(beta.star[-burn]))

cat(" quantiles:\n")

print(quantile(beta.star[-burn], probs = seq(0, 1, 0.025)))

cat(" Acceptance number and rate\n")

N_all=N+Nb

rate.beta=accept.beta/N_all

cat(" overall N: ",N_all," accepted: ",accept.beta," fraction: ",rate.beta,"\n")

cat(" Number of NaN's and rate of NaN's in estimated Metrop-Hast-Fractions\n")

N_all=N+Nb

rate.nan=nan.beta/N_all

cat(" overall N: ",N_all," Nan's: ",nan.beta," fraction: ",rate.nan,"\n\n")

cat("\n\nfinal Gaussian random walk (without burn-in):")

print(length(xsm_exp.star[-burn]))

cat("\nposterior XSM_EXP-Werte (without burn-in)\n")

print(summary(xsm_exp.star[-burn]))

cat(" standard deviation:")

print(sd(xsm_exp.star[-burn]))

cat(" quantiles:\n")

print(quantile(xsm_exp.star[-burn], probs = seq(0, 1, 0.025)))

cat(" Acceptance number and rate\n")

N_all=N+Nb

rate.xsm_exp=accept.xsm_exp/N_all

cat(" overall N: ",N_all," accepted: ",accept.xsm_exp," fraction: ",rate.xsm_exp,"\n")

cat(" Number of NaN's and rate of NaN's in estimated Metrop-Hast-Fractions\n")

N_all=N+Nb

rate.nan=nan.xsm_exp/N_all

cat(" overall N: ",N_all," Nan's: ",nan.xsm_exp," fraction: ",rate.nan,"\n\n")

cat("\n\nfinal Gaussian random walk (without burn-in):")

print(length(xpq_exp.star[-burn]))

cat("\nposterior XPQ_EXP-Werte (without burn-in)\n")

print(summary(xpq_exp.star[-burn]))

cat(" standard deviation:")

print(sd(xpq_exp.star[-burn]))

cat(" quantiles:\n")

print(quantile(xpq_exp.star[-burn], probs = seq(0, 1, 0.025)))

cat(" Acceptance number and rate\n")

N_all=N+Nb

rate.xpq_exp=accept.xpq_exp/N_all

cat(" overall N: ",N_all," accepted: ",accept.xpq_exp," fraction: ",rate.xpq_exp,"\n")

cat(" Number of NaN's and rate of NaN's in estimated Metrop-Hast-Fractions\n")

N_all=N+Nb

rate.nan=nan.xpq_exp/N_all

cat(" overall N: ",N_all," Nan's: ",nan.xpq_exp," fraction: ",rate.nan,"\n\n")

cat("\n\nfinal Gaussian random walk (without burn-in):")

print(length(xsm_nexp.star[-burn]))

cat("\nposterior XSM_NEXP-Werte (without burn-in)\n")

print(summary(xsm_nexp.star[-burn]))

cat(" standard deviation:")

print(sd(xsm_nexp.star[-burn]))

cat(" quantiles:\n")

print(quantile(xsm_nexp.star[-burn], probs = seq(0, 1, 0.025)))

cat(" Acceptance number and rate\n")

N_all=N+Nb

rate.xsm_nexp=accept.xsm_nexp/N_all

cat(" overall N: ",N_all," accepted: ",accept.xsm_nexp," fraction: ",rate.xsm_nexp,"\n")

cat(" Number of NaN's and rate of NaN's in estimated Metrop-Hast-Fractions\n")

N_all=N+Nb

rate.nan=nan.xsm_nexp/N_all

cat(" overall N: ",N_all," Nan's: ",nan.xsm_nexp," fraction: ",rate.nan,"\n\n")

cat("\n\nfinal Gaussian random walk (without burn-in):")

print(length(xpq_nexp.star[-burn]))

cat("\nposterior XPQ_NEXP-Werte (without burn-in)\n")

print(summary(xpq_nexp.star[-burn]))

cat(" standard deviation:")

print(sd(xpq_nexp.star[-burn]))

cat(" quantiles:\n")

print(quantile(xpq_nexp.star[-burn], probs = seq(0, 1, 0.025)))

cat(" Acceptance number and rate\n")

N_all=N+Nb

rate.xpq_nexp=accept.xpq_nexp/N_all

cat(" overall N: ",N_all," accepted: ",accept.xpq_nexp," fraction: ",rate.xpq_nexp,"\n")

cat(" Number of NaN's and rate of NaN's in estimated Metrop-Hast-Fractions\n")

N_all=N+Nb

rate.nan=nan.xpq_nexp/N_all

cat(" overall N: ",N_all," Nan's: ",nan.xpq_nexp," fraction: ",rate.nan,"\n\n")

cat("\n\nfinal Gaussian random walk (without burn-in):")

print(length(bsm.star[-burn]))

cat("\nposterior BSM-Werte (without burn-in)\n")

print(summary(bsm.star[-burn]))

cat(" standard deviation:")

print(sd(bsm.star[-burn]))

cat(" quantiles:\n")

print(quantile(bsm.star[-burn], probs = seq(0, 1, 0.025)))

cat(" Acceptance number and rate\n")

N_all=N+Nb

rate.bsm=accept.bsm/N_all

cat(" overall N: ",N_all," accepted: ",accept.bsm," fraction: ",rate.bsm,"\n")

cat(" Number of NaN's and rate of NaN's in estimated Metrop-Hast-Fractions\n")

N_all=N+Nb

rate.nan=nan.bsm/N_all

cat(" overall N: ",N_all," Nan's: ",nan.bsm," fraction: ",rate.nan,"\n\n")

cat("\n\nfinal Gaussian random walk (without burn-in):")

print(length(bpq.star[-burn]))

cat("\nposterior BPQ-Werte (without burn-in)\n")

print(summary(bpq.star[-burn]))

cat(" standard deviation:")

print(sd(bpq.star[-burn]))

cat(" quantiles:\n")

print(quantile(bpq.star[-burn], probs = seq(0, 1, 0.025)))

cat(" Acceptance number and rate\n")

N_all=N+Nb

rate.bpq=accept.bpq/N_all

cat(" overall N: ",N_all," accepted: ",accept.bpq," fraction: ",rate.bpq,"\n")

cat(" Number of NaN's and rate of NaN's in estimated Metrop-Hast-Fractions\n")

N_all=N+Nb

rate.nan=nan.bpq/N_all

cat(" overall N: ",N_all," Nan's: ",nan.bpq," fraction: ",rate.nan,"\n\n")

# CENTRAL FINDING ABOUT SMR

cat("\n\n******************************************************")

cat("\n\nfinal Gaussian random walk (without burn-in):")

print(length(beta.star[-burn]))

cat("\nposterior SMR (without burn-in)\n")

smr.star=exp(beta.star)

print(summary(smr.star[-burn]))

cat(" standard deviation:")

print(sd(smr.star[-burn]))

cat(" quantiles:\n")

print(quantile(smr.star[-burn], probs = seq(0, 1, 0.025)))

cat("\n\n******************************************************\n\n\n")

jpeg(file="J:/Morfeld/R_work/Metropolis_carbon_black_smoking_prior-exposure_smr_posterior_Analysis_1.jpeg", width =1500, height = 1500, quality=200)

par(mfcol=c(1,1),cex=3,ask=T)

hist(smr.star[-burn],main="posterior SMR, bias adjusted ",xlab=" ")

dev.off()

cat("\n\n")

warnings()

sink() # close output
